# Highly Efficient Selective Hydrogenation of Cinnamaldehyde to Cinnamyl Alcohol over CoRe/TiO_2_ Catalyst

**DOI:** 10.3390/molecules28083336

**Published:** 2023-04-10

**Authors:** Mengting Chen, Yun Wang, Limin Jiang, Yuran Cheng, Yingxin Liu, Zuojun Wei

**Affiliations:** 1 College of Pharmaceutical Science, Zhejiang University of Technology, Hangzhou 310014, China; 2 Key Laboratory of Biomass Chemical Engineering of the Ministry of Education, College of Chemical and Biological Engineering, Zhejiang University, Hangzhou 310027, China

**Keywords:** cinnamaldehyde, cinnamyl alcohol, CoRe bimetallic catalyst, selective hydrogenation, formic acid

## Abstract

Allylic alcohols typically produced through selective hydrogenation of *α*,*β*-unsaturated aldehydes are important intermediates in fine chemical industry, but it is still a challenge to achieve its high selectivity transformation. Herein, we report a series of TiO_2_-supported CoRe bimetallic catalysts for the selective hydrogenation of cinnamaldehyde (CAL) to cinnamyl alcohol (COL) using formic acid (FA) as a hydrogen donor. The resultant catalyst with the optimized Co/Re ratio of 1:1 can achieve an exceptional COL selectivity of 89% with a CAL conversion of 99% under mild conditions of 140 °C for 4 h, and the catalyst can be reused four times without loss of activity. Meanwhile, the Co_1_Re_1_/TiO_2_/FA system was efficient for the selective hydrogenation of various *α*,*β*-unsaturated aldehydes to the corresponding *α*,*β*-unsaturated alcohols. The presence of ReO_x_ on the Co_1_Re_1_/TiO_2_ catalyst surface was advantageous to the adsorption of C=O, and the ultrafine Co nanoparticles provided abundant hydrogenation active sites for the selective hydrogenation. Moreover, FA as a hydrogen donor improved the selectivity to *α*,*β*-unsaturated alcohols.

## 1. Introduction

Selective hydrogenation of *α*,*β*-unsaturated aldehydes to unsaturated alcohols is an important process used to obtain a great deal of valuable chemicals [1,2,3,4]. Cinnamaldehyde (CAL), a typical *α*,*β*-unsaturated aldehyde, could be selectively hydrogenated to generate cinnamyl alcohol (COL), which is regarded as one of the most promising building blocks in pharmaceutical, agrochemical and fragrance industries [5,6].

Generally, the selective hydrogenation of CAL leads to the reduction of different functional groups, including C=O and C=C, and produces COL, 3-phenylpropionaldehyde (HCAL) and 3-phenylpropanol (HCOL) [7,8,9,10]. Owing to a higher binding energy of C=O bonds than C=C bonds (715 vs. 615 KJ·mol^−1^), the hydrogenation of C=O bonds is more unfavorable in thermodynamics [11,12,13]. Therefore, it is essential to develop high-performance catalysts to improve the selectivity hydrogenation of C=O bonds and avoid the hydrogenation of C=C bonds.

Supported metal nanoparticle catalysts have been widely used in the industry due to their merits of easy separation and recovery [7,14]. The radial expansion of a metal _D_-bandwidth and d orbital is related to the selectivity of products (including COL, HCAL and HCOL) [15]. Metal with a small _D_-bandwidth (such as Ni) is conducive to the formation of HCAL [16], while some noble metals with a relatively large _D_-bandwidth (such as Ru, Ir, Au and Pt) can be used as catalysts for the synthesis of COL [17,18,19,20,21]. However, the high cost and rarity of noble metal catalysts hinder their industrial application. Non-noble Co has shown potential in the selective hydrogenation of CAL to COL due to its larger _D_-bandwidth and low price. For example, Zhang et al. [22] investigated the performance of the Co/ZSM-5 catalyst for the hydrogenation of CAL to COL at 90 °C and 20 bar H_2_ for 6 h, with a 72.7% conversion of CAL and a 78.5% selectivity to COL. In another work using Co/ZSM-5 as the catalyst, a maximum COL yield of 61.9% was achieved at 100 °C and 20 bar H_2_ [23].

However, monometallic Co catalysts have noticeable issues such as poor catalytic activity and high metal dosage. It is widely believed that the introduction of a second metal is an effective way to enhance the catalytic properties of Co nanoparticles, although the catalytic mechanism of the bimetallic catalysts is far from clear [24]. Adjusting the metal–metal/metallic oxide interactions in the catalyst could improve the morphology of dispersed metals and result in electron transfer, thus enhancing the charge density of the active metal and affecting the adsorption/desorption of C=O or C=C bonds on CAL [5,25]. For instance, the CoGa_3_/MgO·Al_2_O_3_-LDH catalyst gave 96% COL selectivity in the hydrogenation of CAL at 100 °C and 20 bar H_2_ for 8 h, which was significantly higher than the monometallic Co catalyst (42%) [26]. CoPt/Fe_3_O_4_ showed excellent catalytic performance under the conditions of 160 °C and 30 bar H_2_, with a CAL conversion of 95% and a COL yield of 84% [27].

Another factor affecting the selectivity of COL in CAL hydrogenation is hydrogen donors. When using molecular hydrogen, a high H_2_ pressure is usually needed in the selective hydrogenation of CAL to COL. However, high H_2_ pressure requires specialized transportation and handling, which is deemed to be unsafe [28]. In addition, the different phases of H_2_ and substrates increase the contact time, thus reducing the reaction efficiency caused by the transport phenomenon [29]. By comparison, the selective hydrogenation by replacing traditional hydrogen with hydrogen donors such as alcohols [30,31,32], formic acid (FA) [33,34] and silanes [35,36] offers a green, safe, sustainable and atomic economic process. For example, Butt et al. [34] reported that a COL yield of 73% was obtained in the hydrogenation of CAL over a AuNPore catalyst using Et_3_SiH as a hydrogen donor at 70 °C for 24 h. When using FA as a hydrogen donor, a COL yield of 97% was achieved over a AuNPore catalyst at 90 °C for 22 h [35]. Herein, we prepared a series of TiO_2_-supported CoRe bimetallic catalysts for the hydrogenation of CAL to COL under mild conditions using FA as an effective hydrogen donor, and we further extended the hydrogenation of various *α*,*β*-unsaturated aldehydes to *α*,*β*-unsaturated alcohols. The choice of Re as the second component is mainly due to the following two considerations. Firstly, high valence Re (+7) can be easily reduced to ReO_x_ (mainly Re (+4) and Re (+6)), which has many oxygen vacancies, and is conducive to the preferential adsorption of the C=O group on CAL [24,37]. Secondly, ReO_x_ has a positive effect on the stability of metal nanoparticles [38], thus reducing the aggregation and leakage of Co during the reaction [39,40]. The structure–activity relationship was analyzed by N_2_ adsorption–desorption, CO chemisorption, TEM and XPS characterizations. In addition, the effects of the reaction parameters and the stability of the green catalytic system were investigated. Finally, the possible reaction mechanism was proposed. The main purpose here is to develop an efficient methodology for the selective hydrogenation of *α*,*β*-unsaturated aldehydes to *α*,*β*-unsaturated alcohols.

## 2. Results and Discussions

### 2.1. Catalytic Activity Test

**Figure 1:**
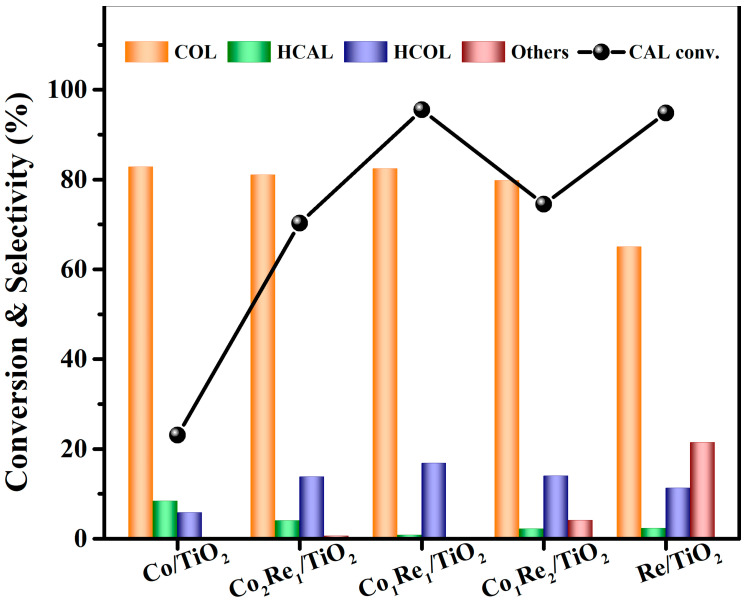
Hydrogenation of CAL to COL over various CoRe/TiO_2_ catalysts. Reaction conditions: 3 mmol CAL, 80 mg catalyst, 10 mL isopropanol, 160 °C, 12 h. Others: allylbenzene, isopropenylbenzene, 1,1′-(1,5-hexadiene-1,6-diyl)bisbenzene and other unknown by-products.

The selective hydrogenation of CAL to COL was firstly investigated over the Co/TiO_2_, Re/TiO_2_ and Co_x_Re_y_/TiO_2_ catalysts (x:y varied from 2:1 to 1:2) using isopropanol as both the solvent and hydrogen donor at 160 °C for 12 h, and the results are shown in Figure 1. It can be seen that the monometallic Co/TiO_2_ catalyst showed a low conversion (23%) of CAL, although it gave a high selectivity (83%) to COL. The addition of Re obviously improved the conversion of CAL, which was consistent with the previous report that introducing the hydrophilic metal Re to the catalyst is beneficial for the adsorption of the C=O bond, thus improving the catalytic performance of the catalyst [41]. The maximum CAL conversion of 96% with a COL selectivity of 82% was achieved over the Co_1_Re_1_/TiO_2_ catalyst, better than the Co_1_Mo_1_/TiO_2_, Co_1_Ce_1_/TiO_2_, Co_1_Zr_1_/TiO_2_ catalysts and the Co_1_Re_1_ catalyst on other supports (SiO_2_, ZrO_2_, γ-Al_2_O_3_ and ZSM-5, Table S1, in the Supplementary Materials). These phenomena might be attributed to the strong adsorption of TiO_2_ and ReO_x_ on exposed the C=O group [42], which improves the diffusion of the substrate and accelerates the hydrogenation of CAL [43].

### 2.2. Catalyst Characterization

The structural parameters of the samples are summarized in Table 1. Deposition of relatively low Co and Re contents on TiO_2_ only slightly influenced its specific surface area, and the S_BET_ values of all catalysts are close to TiO_2_ and remain near 50 m^2^·g^−1^. On the other hand, the average pore diameter D_p_ of metal-supported catalysts Co/TiO_2_, Re/TiO_2_ and Co_1_Re_1_/TiO_2_ were larger than TiO_2_, and the pore volumes V_p_ of them were reduced, which could be explained by partial blocking of the narrowest pores by the metallic phase, indicating that the metals were embedded in the carrier pores [44].

**Figure 2:**
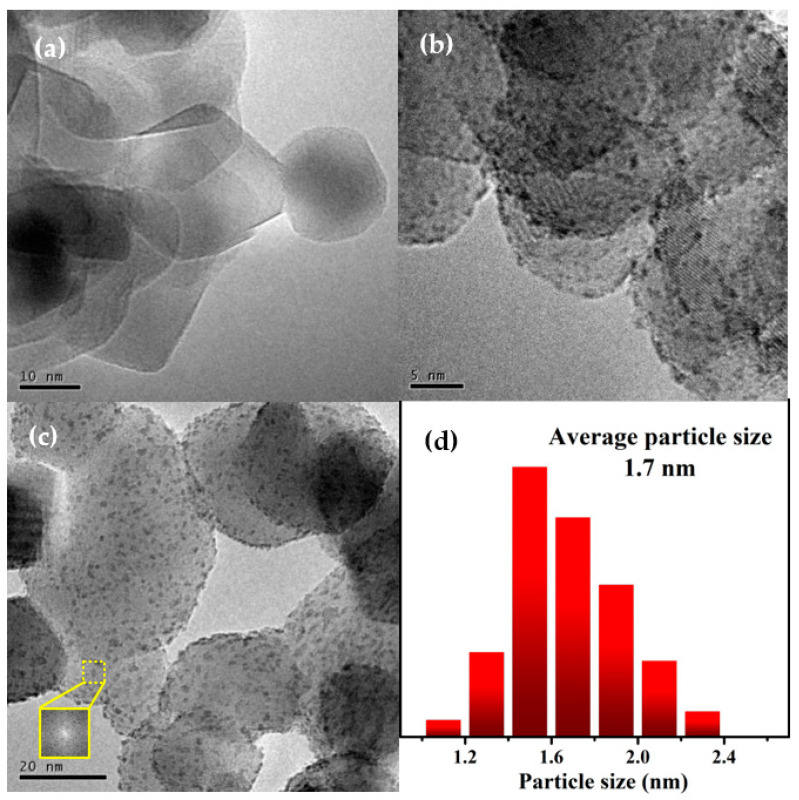
TEM images of (**a**) Co/TiO_2_ and (**b**,**c**) Co_1_Re_1_/TiO_2_ catalysts and (**d**) particle size distribution of Co_1_Re_1_/TiO_2_.

The morphologies of Co/TiO_2_ and Co_1_Re_1_/TiO_2_ catalysts were characterized by TEM. As illustrated in Figure 2a, no clear Co nanoparticles were observed on the Co/TiO_2_ catalyst, which is in agreement with Cheng’s work [45]. As seen from Figure 2c, no diffraction spot was observed in the fast Fourier transformation (FFT) image of the CoRe particles, which is similar to our earlier work [46], implying its amorphous structure. The size-distribution histogram substantiates that the average size of the CoRe nanoparticles is about 1.7 nm (Figure 2d), which is similar to the size (1.8 nm) measured by CO chemisorption, and is much smaller than monometallic Co (8.1 nm, Table 1). This observation was further demonstrated by the results of Co dispersion measured by CO chemisorption (Table 1). The dispersion of Co on Co_1_Re_1_/TiO_2_ is much higher than that of Co/TiO_2_. These phenomena manifest that the introduction of the second metal Re significantly reduced the particle size of the Co nanoparticles and improved Co dispersion, thus providing more hydrogenation active sites for the selective hydrogenation of CAL.

**Figure 3:**
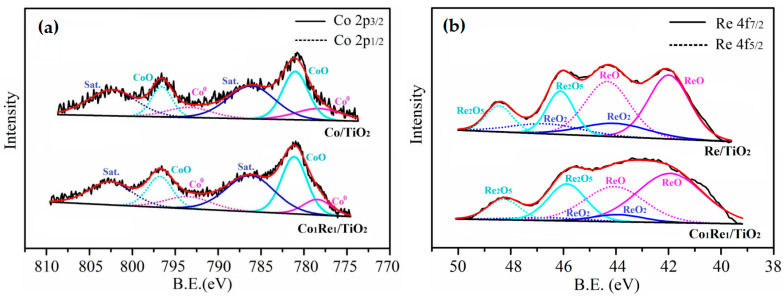
XPS spectra of (**a**) Co 2p and (**b**) Re 4f of the reduced catalysts Co/TiO_2_, Re/TiO_2_ and Co_1_Re_1_/TiO_2_.

The chemical state and surface composition of Co/TiO_2_, Re_1_/TiO_2_ and Co_1_Re_1_/TiO_2_ catalysts were assessed by XPS (Figure 3), and the calculated abundances of different surface Co and Re species are summarized in Table S3. As shown in Figure 3a, the peaks in the Co 2p spectrum of Co/TiO_2_ at 778.2 and 793.4 eV are assigned to Co^0^, the peaks at 781.0 and 796.8 eV are attributed to CoO, and the peaks at 796.1 and 802.5 eV are satellite peaks [47]. The observation of CoO was due to the oxidation of surface metallic Co nanoparticles in the air. As shown in Figure 3b, the binding energy in the range of 39–50 eV belonged to the Re 4f region, which was deconvoluted into doublet peaks for 4f_5/2_ and 4f_7/2_ orbits, implying the presence of ReO (4f_5/2_ = 44.2 eV, 4f_7/2_ = 41.9 eV), ReO_2_ (4f_5/2_ = 46.3 eV, 4f_7/2_ = 44.0 eV), and Re_2_O_5_ (4f_5/2_ = 48.3 eV, 4f_7/2_ = 46.0 eV) [48] in Re/TiO_2_ with an atomic ratio of 65:16:19 (Table S2), and no metallic Re was detected, which was contributed to the high affinity of Re for oxygen [49]. Compared to their monometallic counterparts, the content of ReO in Co_1_Re_1_/TiO_2_ significantly increased to 71%, which may promote the spillover of hydrogen on its surface and therefore be beneficial to the hydrogenation process [46].

### 2.3. Effects of Reaction Conditions

As mentioned in Figure 1, using isopropanol as a hydrogen donor over the Co_1_Re_1_/TiO_2_ catalyst can cause a high yield of COL to be obtained. However, a higher temperature (160 °C) and a longer reaction time (12 h) were required, and a great amount of HCOL (17%) was generated, thus decreasing COL selectivity. As shown in Table S3 (entry 4), compared to other common hydrogen donors (isopropanol, triethyl silicane and ammonium formate), when using FA as the hydrogen donor, a high COL yield could be achieved under mild reaction conditions in the inert solvent tetrahydrofuran (THF). Meanwhile, FA is green, sustainable, atom economical and easy to operate. Therefore, it is a good choice of hydrogen donor for the selective hydrogenation of CAL to COL. Thus, we used FA as a hydrogen donor to further study the hydrogenation of CAL to COL. Based on the results in Table 2, the combination of FA and triethylamine (NEt_3_) was found to be essential for achieving high activity and selectivity in the hydrogenation of CAL to COL over Co_1_Re_1_/TiO_2_, and a CAL conversion of 99% and a COL selectivity of 89% were obtained at a FA:NEt_3_ molar ratio of 1:1 (Table 2, entry 2), which displayed obvious advantages over molecular hydrogen (Table 2, entry 5). The addition of NEt_3_ to provide basic sites can substantially facilitate the crucial FA deprotonation process, which appears to be a key factor for achieving high activity of CAL hydrogenation to COL [50]. Too much or too little amounts of NEt_3_ in the reaction system, however, led to a decrease in the selectivity of COL (Table 2, entries 3 and 4). In addition, the CAL:FA molar ratio also affected the reactivity of the hydrogenation of CAL to COL, as shown in Table 3. Low amounts of FA led to the deficiency of active hydrogen and to the decrease in the conversion of CAL, while excess FA led to an increase in by-products, and the optimal molar ratio of CAL:FA was 1:2 (Table 3, entry 2).

**Figure 4:**
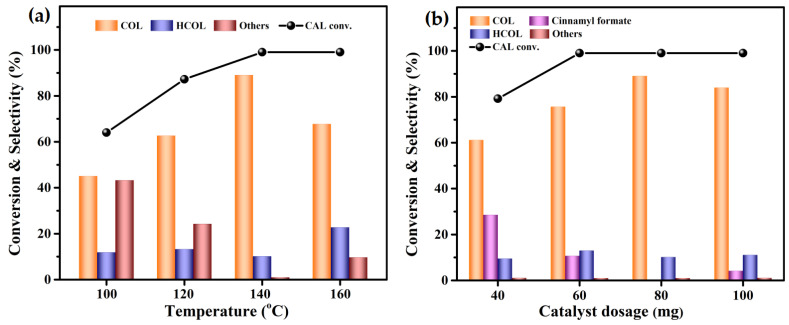
Effect of (**a**) reaction temperature; (**b**) Co_1_Re_1_/TiO_2_ catalyst dosage for the hydrogenation of CAL to COL. Reaction conditions: 3 mmol CAL, CAL:FA:NEt_3_ = 1:2:2, 10 mL THF, 80 mg Co_1_Re_1_/TiO_2_ catalyst, 140 °C, 4 h. Others: allylbenzene, isopropenylbenzene, 1,1′-(1,5-hexadiene-1,6-diyl)bisbenzene and other unknown by-products.

The effects of the reaction temperature and catalyst dosage for the hydrogenation of CAL to COL were investigated over Co_1_Re_1_/TiO_2_, and the results are shown in Figure 4. Figure 4a shows the results of the hydrogenation of CAL at varied temperatures (from 100 to 160 °C). During the reaction, no HCAL was detected. The catalyst showed low activity for the reaction at a low temperature of 100 °C. Increasing the reaction temperature to 140 °C achieved a remarkable increase in the catalytic performance. However, the excessive reaction temperature (160 °C) led to a significant increase in the excessive hydrogenation of HCOL and other by-products. Thus, 140 °C was the appropriate reaction temperature for CAL-selective hydrogenation to COL. Fixing the reaction temperature at 140 °C, the hydrogenation of CAL was carried out over the Co_1_Re_1_/TiO_2_ catalyst at a dosage in the range from 40 to 100 mg, and the results are shown in Figure 4b. It can be seen that the yield of COL was significantly improved with the increase in catalyst dosage from 40 to 80 mg. However, an excessive catalyst dosage reduced the selectivity of COL increased the excessive hydrogenation by-product HCOL and other by-products. As expected, our rationally designed Co_1_Re_1_/TiO_2_ catalyst exhibited higher activity and selectivity for the hydrogenation of CAL to COL compared with TiO_2_, Co/TiO_2_, and Re/TiO_2_ catalysts (Table S4), and it showed significant advantages compared with the relevant literature (Table S5) [24,27,30,32,33,34,35,45,51,52,53,54,55,56,57,58,59,60,61]. The results of the selective hydrogenation of CAL to COL suggest that the synergistic effect among the TiO_2_ support and Co and Re metals is the main reason for the enhanced performance of the Co_1_Re_1_/TiO_2_ catalyst.

**Figure 5:**
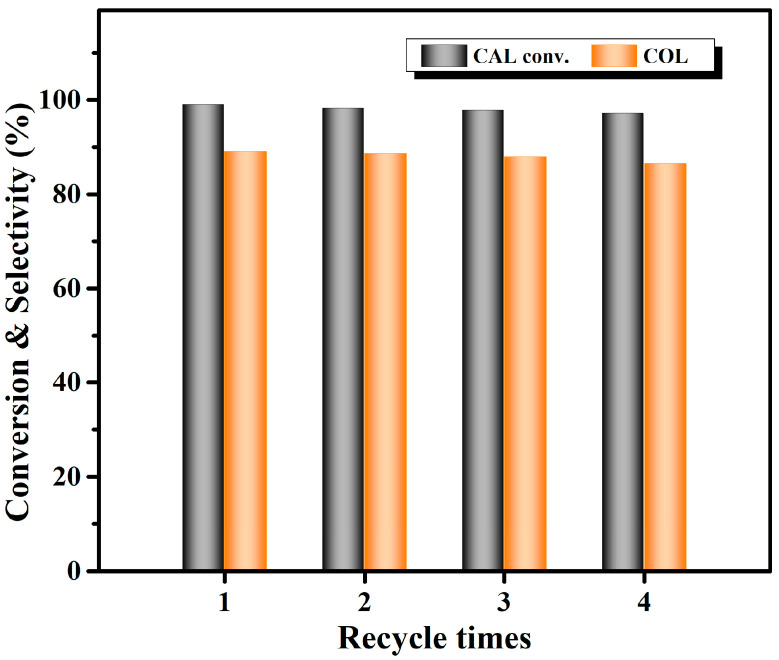
Recyclability of Co_1_Re_1_/TiO_2_ catalyst. Reaction conditions: 3 mmol CAL, CAL:FA:Net_3_ = 1:2:2, 10 mL THF, 80 mg catalyst, 140 °C, 4 h.

Subsequently, we investigated the stability of the Co_1_Re_1_/TiO_2_ catalyst for the hydrogenation of CAL to COL. During each cycle, after a complete reaction at 140 °C for 4 h, the catalyst was centrifuged, washed with THF for five times, and reused for the next runs. As shown in Figure 5, the Co_1_Re_1_/TiO_2_ catalyst kept its good performance during recycling, the conversion of CAL was 97%, and the selectivity of COL was 87% after four runs. The XRD pattern of the spent catalyst showed no noticeable morphology changes compared with the fresh one (Figure S1). Moreover, the comparable metal contents in the fresh and spent catalysts determined by ICP-OES indicated no obvious metal leaching (Table S6). These results suggest that the Co_1_Re_1_/TiO_2_ catalyst has excellent stability for the hydrogenation of CAL to COL.

In addition, the applicability of the Co_1_Re_1_/TiO_2_/FA system was tested for the selective hydrogenation of various *α*,*β*-unsaturated aldehydes to the corresponding *α*,*β*-unsaturated alcohols under the optimized reaction conditions, except for the reaction time, which was adjusted to obtain high yields. The results are listed in Table 4. Similar to COL, *α*-methyl cinnamaldehyde also achieved high conversion (99%, entry 1) and selectivity (86%), and the remaining 14% selectivity was attributed to simultaneous hydrogenation of C=C and C=O by-product. Moreover, aliphatic crotonaldehyde and citral could be converted well, and the target products were offered with selectivities of 74% and 96%, respectively (entries 2, 3). It is clear that substituent groups greatly affected the performance of the Co_1_Re_1_/TiO_2_ catalyst. In general, the selectivity to *α*,*β*-unsaturated alcohols is closely related to the steric prohibition of the substituent groups. The more steric prohibition of C=C by the substitutes at γ-carbon, the higher the C=O selectivity, which is in good agreement with the literature [62]. Furthermore, a high conversion of 99% was obtained after the hydrogenation of cycloaliphatic isophorone (entry 4), with a product selectivity of 84% accompanied by excessive hydrogenation by-product of 16%. These results well verify that the Co_1_Re_1_/TiO_2_/FA system is effective for the selective hydrogenation of *α*,*β*-unsaturated aldehydes to *α*,*β*-unsaturated alcohols.

**Figure 6:**
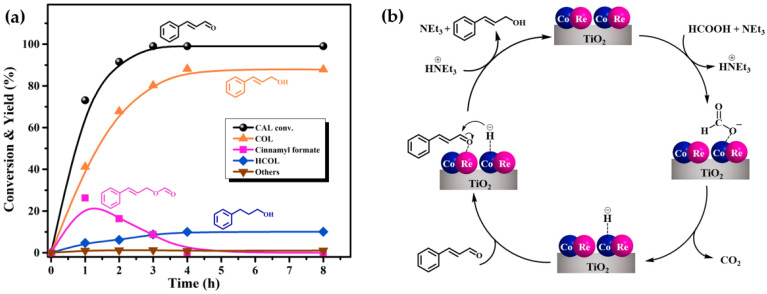
(**a**) Time courses of the hydrogenation of CAL to COL over Co_1_Re_1_/TiO_2_. Reaction conditions: 3 mmol CAL, CAL:FA:NEt_3_ = 1:2:2, 10 mL THF, 80 mg Co_1_Re_1_/TiO_2_ catalyst, 140 °C. Others: allylbenzene, isopropenylbenzene, 1,1′-(1,5-hexadiene-1,6-diyl)bisbenzene and other unknown by-products. (**b**) The proposed mechanism for the hydrogenation of CAL to COL over Co_1_Re_1_/TiO_2_.

To speculate the possible reaction pathway, the time course experiments of the hydrogenation of CAL over the Co_1_Re_1_/TiO_2_ catalyst was carried out under optimal reaction conditions (Figure 6a). As shown in Figure 6a, CAL was rapidly converted (73%), and a 42% yield of COL was achieved at the initial 1 h, accompanied by 26% of cinnamyl formate and 5% of HCOL. Under the weak alkaline condition of NEt_3_, cinnamyl formate could be easily formed through esterification of COL with formic acid [63], which then smoothly decreased and disappeared within 4 h with the decomposition of formic acid. CAL was almost completely converted at 4 h, and COL and HCOL were offered with yields of 88% and 10%, respectively. It is recognized that the decomposition mechanism of FA in the presence of NEt_3_ is as follows [34,64,65] NEt_3_ acts as a proton scavenger to facilitate the O-H bond cleavage, thus forming metal–formate species during the initial step of the reaction. Then metal–formate produces molecular hydrogen through a *β*-elimination pathway. This hydrogen desorption step is irreversible, indicating that it is feasible to use renewable FA as a convenient hydrogen donor instead of molecular H_2_ for sustainable and green organic synthesis. The proposed mechanism is shown in Figure 6b. The Co_1_Re_1_/TiO_2_ catalyst and FA produce metal–formate species in the presence of NEt_3_, which are then decomposed to generate metal–hydride species and CO_2_. Meanwhile, the C=O bond on CAL is adsorbed on the ReO_x_ species and is coordinated with metal–hydride. Finally, it is neutralized with ^+^HNEt_3_ to form COL, thereby realizing the regeneration of the Co_1_Re_1_/TiO_2_ catalyst and NEt_3_.

## 3. Experimental Section

### 3.1. Materials

Co(NO_3_)_2_·6H_2_O (99%) was purchased from Shanghai Jiuling Chemical Co., Ltd. (Shanghai, China). NH_4_ReO_4_ (99%) was purchased from Shanghai Macklin Biochemical Co., Ltd. (Shanghai, China). Isopropanol (99.5%), tetrahydrofuran (99.5%) and formic acid (99%) were purchased from Yonghua Chemical Technology Co., Ltd. (Suzhou, China). Triethylamine (99.5%) was purchased from Sinopharm Chemical Reagent Co., Ltd. (Shanghai, China). Other chemicals were purchased from Aladdin Reagent Co., Ltd. (Shanghai, China). All the chemicals used in this work were analytical reagents and were used without further purification.

### 3.2. Preparation of Various CoRe/TiO_2_ Catalysts

A series of TiO_2_-supported CoRe catalysts with variable Co:Re molar ratios (Co:Re = 2:1, 1:1 or 1:2) were prepared by an incipient wetness impregnation method. The loading of Co in the catalysts was kept at 2 wt%. Taking the CoRe/TiO_2_ catalyst with a Co:Re molar ratio of 1:1 as an example, the preparation method was as follows: firstly, TiO_2_ was calcined at 500 °C for 4 h to remove the impurities prior to impregnation of the metal precursor. Then, 0.0998 g of Co(NO_3_)_2_·6H_2_O and 0.0920 g of NH_4_ReO_4_ were dissolved in ca. 1 mL deionized H_2_O. An appropriate amount of TiO_2_ (ca. 0.98 g) was slowly added to the aqueous solution under ultrasound. After being impregnated at room temperature for 24 h, the mixture was dried at 110 °C overnight and finally reduced at 500 °C in a tubular furnace under hydrogen flow for 3 h to obtain the target catalyst, which was denoted as Co_1_Re_1_/TiO_2_. Two monometallic catalysts 2 wt% Co/TiO_2_ and 2 wt% Re/TiO_2_ were prepared by using the same method for comparison. The information on the content of Co and Re in the corresponding catalyst is described in Table S7.

### 3.3. Catalyst Characterization

Brunauer–Emmett–Teller (BET) surface areas and pore structures of the catalyst samples were measured by pulsed N_2_ adsorption–desorption method at −196 °C using Micromeritics ASAP 2460 analyzer. Before N_2_ physisorption, the samples were degassed under vacuum at 250 °C for 3 h. Transmission electron microscopy (TEM) images were obtained using a Tecnai G2 F30 S-Twin instrument (FEI Co., Columbia, SC, USA). The particle size distribution of metal nanoparticles in the sample was determined by measuring approximately 100 particles randomly selected from the TEM micrographs. The metal dispersion and particle size were measured by CO chemisorption at 35 °C using a Micromeritics ASAP 2020 system. X-ray diffraction (XRD) was conducted using an X’ Pert PRO X-ray diffractometer equipped with Cu Kα radiation at 40 kV and 40 mA (λ = 0.15405 nm). Samples were scanned from 20° to 80° with a scanning rate of 4°·min^−1^ and a step size of 0.02°. The content of the metals was measured by inductively coupled plasma-optical emission spectroscopy (ICP-OES) using a Thermo Fisher iCAP PRO. X-ray photoelectron spectroscopy (XPS) spectra were obtained using an Escalab Mark II X-ray spectrometer (VG Co., Manchester, UK) equipped with a magnesium anode (Mg Kα = 1253.6 eV). Energy corrections were performed using a C 1s peak of the pollutant carbon at 284.8 eV.

### 3.4. Catalytic Performance

The catalytic hydrogenation of *α*,*β*-unsaturated aldehydes to *α*,*β*-unsaturated alcohols was performed in a 25 mL stainless-steel autoclave equipped with magnetic stirring. In a typical experiment, 3 mmol of *α*,*β*-unsaturated aldehyde, 6 mmol of FA, 6 mmol of NEt_3_, 80 mg of catalyst and 10 mL of THF were added into the reactor. The reactor was sealed and purged with N_2_ five times. The autoclave was then heated to the required temperature and kept at this temperature for the required time under the continuous stirring speed of 1000 rpm. After the reaction, the autoclave was quickly cooled to room temperature, and the reaction products were separated from the catalyst by centrifugation and quantitatively analyzed with an Agilent 7890 A gas chromatographer equipped with an HP-5 capillary column (30.0 m × 0.32 mm × 0.25 μm) and a flame ionization detector (FID) using *n*-dodecane as an internal standard. The conformation of the products was performed on an Agilent 6890 GC system coupled to a mass spectrometer equipped with an Agilent 5973 quadrupole mass analyzer. CAL conversion and selectivity and the yield of the products were calculated by the following equations.

(1) $$\mathrm{Conversion}\;(\%)=\frac{moles\;of\;substrate\;consumed}{moles\;of\;initial\;substrate}\;\times 100\%$$

(2) $$\mathrm{Selectivity}\;(\%)=\frac{moles\;of\;desired\;product\;formed}{moles\;of\;substrate\;consumed}\;\times 100\%$$

(3) Yield (%)=Conversion ×Selectivity ×100%

## 4. Conclusions

In summary, CoRe bimetallic catalysts supported on TiO_2_ were achieved and were first reported in the selective hydrogenation of CAL to COL using FA as a hydrogen donor to replace the traditional molecular hydrogen. Especially, the Co_1_Re_1_/TiO_2_ catalyst performed excellent activity, selectivity and stability, with a 99% conversion of CAL and 89% COL selectivity, and no obvious deactivation was observed after using it four times. Under similar reaction conditions, using *α*-methyl cinnamaldehyde, crotonaldehyde, citral and isophorone as feedstocks, high conversions and excellent selectivities to allylic alcohols were also achieved. The technique established in this work provides a green, mild and efficient process for the selective hydrogenation of *α*,*β*-unsaturated aldehydes to allylic alcohols.

## Figures and Tables

**Table 1:** Textural properties of TiO_2_, Co/TiO_2_, Re/TiO_2_ and Co_1_Re_1_/TiO_2_.

Sample	d_TEM_ (nm)	d_Co_^a^ (nm)	Dispersion of Co ^a^ (%)	S_BET_ (m^2^·g^−1^)	D_p_^b^ (nm)	V_p_^b^ (cm^3^·g^−1^)
TiO_2_	/	/	/	52.8	11.3	0.15
Co/TiO_2_	/	8.1	12.3	52.9	28.9	0.41
Co_1_Re_1_/TiO_2_	1.7	1.8	55.7	48.5	24.5	0.33
Re/TiO_2_	/	/	/	50.7	27.5	0.37

^a^ Determined by CO chemisorption. ^b^ The pore size and pore volumes were derived from the adsorption branches of isotherms by using the BJH model.

**Table 2:** Hydrogenation of CAL to COL using different hydrogen donors.

Entry	Hydrogen Donor	Molar Ratio (FA:NEt_3_)	Conv. (%)	Sel. (%)			
				COL	HCAL	HCOL	Others
1	FA	/	64	29	4	3	64
2	FA	1:1	99	89	/	10	1
3	FA	2:5	99	72	/	28	/
4	FA	5:2	99	80	/	20	/
5 ^a^	Hydrogen	/	57	17	42	25	16

Reaction conditions: 3 mmol CAL, CAL:FA = 1:2, 10 mL THF, 80 mg Co_1_Re_1_/TiO_2_ catalyst, 140 °C, 4 h. ^a^ 2.5 MPa H_2_. Others: allylbenzene, isopropenylbenzene, 1,1′-(1,5-hexadiene-1,6-diyl)bisbenzene and other unknown by-products.

**Table 3:** Effect of CAL:FA molar ratio on the hydrogenation of CAL to COL.

Entry	Molar Ratio (CAL:FA)	Conv. (%)	Sel. (%)			
			COL	HCAL	HCOL	Others
1	1:1.5	92	89	/	11	/
2	1:2	99	89	/	10	1
3	1:3	94	60	/	7	33

Reaction conditions: 3 mmol CAL, FA:NEt_3_ = 1:1, 10 mL THF, 80 mg Co_1_Re_1_/TiO_2_ catalyst, 140 °C, 4 h. Others: allylbenzene, isopropenylbenzene, 1,1′-(1,5-hexadiene-1,6-diyl)bisbenzene and other unknown by-products.

**Table 4:** Hydrogenation of various *α*,*β*-unsaturated aldehydes over the Co_1_Re_1_/TiO_2_ catalyst.

Entry	Substrate	Time (h)	Conv. (%)	Sel. (%)	
1	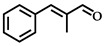	5	99	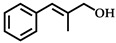 (86)	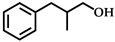 (14)
2	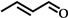	8	93	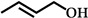 (74)	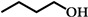 (26)
3	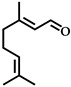	8	92	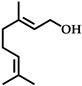 (96)	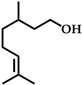 (5)
4	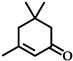	12	99	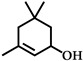 (84)	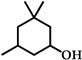 (16)

Reaction conditions: 3 mmol substrate, substrate:FA:NEt_3_ = 1:2:2, 10 mL THF, 80 mg Co_1_Re_1_/TiO_2_ catalyst, 140 °C.

## Data Availability

The data presented in this study are available on request from the corresponding author.

## Supplementary Materials

The following supporting information can be downloaded at: https://www.mdpi.com/article/10.3390/molecules28083336/s1, Table S1: Hydrogenation of CAL over different Co-based catalysts; Table S2: Co 2p and Re 4f dispersion on different catalysts; Table S3: Hydrogenation of CAL to COL using different hydrogen donors; Table S4: Hydrogenation of CAL to COL over various catalysts using formic acid as the hydrogen donor; Table S5: Hydrogenation of CAL to COL over various catalytic systems; Table S6: Metal contents in fresh and spent Co_1_Re_1_/TiO_2_ catalyst measured by ICP-OES; Table S7: Labels and calculated metal contents of catalysts; Figure S1: XRD patterns of fresh and spent Co_1_Re_1_/TiO_2_; Figure S2: GC/MS spectra of substrate and products in the hydrogenation of COL; Figure S3: GC/MS spectra of the main products in the hydrogenation of other *α*,*β*-unsaturated aldehydes. References [24,27,30,32,33,33,34,35,45,51,52,53,54,55,56,57,58,59,60,61] are cited in the supplementary materials.
