# Supplementary material for: Highly Efficient Selective Hydrogenation of Cinnamaldehyde to Cinnamyl Alcohol over CoRe/TiO2 Catalyst
Source: Molecules. 2023 Apr 10;28(8):3336. doi: 10.3390/molecules28083336 (PMC10142762; doi:10.3390/molecules28083336)
Supplement: Supplementary file 1 [file molecules-28-03336-s001.zip › molecules-2312033-supplementary.pdf]

Supporting Information for

Highly efficient selective hydrogenation of cinnamaldehyde to cinnamyl alcohol over CoRe/TiO_2 catalyst

Mengting Chen[1,†], Yun Wang[1,†], Limin Jiang[1], Yuran Cheng[2], Yingxin Liu[1,*], Zuojun Wei[2,*]

[1] *College of Pharmaceutical Science, Zhejiang University of Technology, Hangzhou 310014, China*

[2] *Key Laboratory of Biomass Chemical Engineering of the Ministry of Education, College of Chemical and Biological Engineering, Zhejiang University, Hangzhou 310027, China*

* Corresponding authors

E-mail address: yxliu@zjut.edu.cn (Y.X. Liu), weizuojun@zju.edu.cn (Z.J. Wei).

[†] These authors contributed equally to this work.

Table S1. Hydrogenation of CAL over different Co-based catalysts.

Entry	Catalyst	Conv. (%)	Sel. (%)			
			COL	HCAL	HCOL	Others
1	Co_1Mo_1/TiO_2	38	75	12	10	3
2	Co_1Ce_1/TiO_2	41	84	6	10	/
3	Co_1Zr_1/TiO_2	37	88	/	/	12
4	Co_1Re_1/TiO_2	96	82	1	17	/
5	Co_1Re_1/SiO_2	18	48	12	8	32
6	Co_1Re_1/ZrO_2	24	13	17	3	67
7	$Co_1Re_1/\gamma\text{-}Al_2O_3$	64	56	18	5	21
8	Co_1Re_1/ZSM-5	/	/	/	/	/

Reaction conditions: 3 mmol CAL, 10 mL isopropanol, 80 mg catalyst, 160 °C, 12 h. Others: allylbenzene, isopropenylbenzene, 1,1'-(1,5-hexadiene-1,6-diyl)bisbenzene and other unknown by-products.

Table S2. Co 2p and Re 4f dispersion on different catalysts.

Catalyst	Elemental species	Binding energy (eV)	Content
Re/TiO_2	ReO $4f_{7/2}$	41.9	65%
	ReO_2 $4f_{7/2}$	44.0	16%
	Re_2O_5 $4f_{7/2}$	46.0	19%
	ReO $4f_{5/2}$	44.2	
	ReO_2 $4f_{5/2}$	46.3	
	Re_2O_5 $4f_{5/2}$	48.3	
Co/TiO_2	Co $2p_{3/2}$	778.2	17%
	CoO $2p_{3/2}$	781.0	39%
	CoO $2p_{3/2}$ shakeup	796.1	44%
	Co $2p_{1/2}$	793.4	
	CoO $2p_{1/2}$	796.8	
	CoO $2p_{1/2}$ shakeup	802.5	
Co_1Re_1/TiO_2	ReO $4f_{7/2}$	41.7	71%
	ReO_2 $4f_{7/2}$	43.8	6%
	Re_2O_5 $4f_{7/2}$	45.8	23%
	ReO $4f_{5/2}$	44.0	
	ReO_2 $4f_{5/2}$	46.1	
	Re_2O_5 $4f_{5/2}$	48.1	
	Co $2p_{3/2}$	778.4	16%
	CoO $2p_{3/2}$	781.2	39%
	CoO $2p_{3/2}$ shakeup	796.3	45%
	Co $2p_{1/2}$	793.6	
	CoO $2p_{1/2}$	797.0	
	CoO $2p_{1/2}$ shakeup	802.7	

Table S3. Hydrogenation of CAL to COL using different hydrogen donors.

Entry	Hydrogen donor	Reaction conditions	Conv. (%)	Sel. (%)			
				COL	HCAL	HCOL	Others
1[a]	Isopropanol	160 °C, 12 h	96	82	1	17	/
2	Triethyl silicane	140 °C, 4 h	34	78	2	19	1
3	Ammonium formate	140 °C, 4 h	94	58	/	22	20
4[b]	Formic acid	140 °C, 4 h	99	89	/	10	1

Reaction conditions: 3 mmol CAL, CAL:hydrogen donor = 1:2, 10 mL THF, 80 mg Co_1Re_1/TiO_2 catalyst. [a]10 mL isopropanol. [b]CAL:FA:NEt_3 = 1:2:2. Others: cinnamyl formate, allylbenzene, isopropenylbenzene, 1,1'-(1,5-hexadiene-1,6-diyl)bisbenzene and other unknown by-products.

Table S4. Hydrogenation of CAL to COL over various catalysts using formic acid as the hydrogen donor.

Entry	Catalyst	Conv. (%)	Sel. (%)			
			COL	HCAL	HCOL	Others
1	TiO_2	/	/	/	/	/
2	Co/TiO_2	26	93	2	5	/
3	Re/TiO_2	98	74	/	8	18
4	Co_1Re_1/TiO_2	99	89	/	10	1

Reaction conditions: 3 mmol CAL, CAL:FA:NEt_3 = 1:2:2, 10 mL THF, 80 mg catalyst, 140 °C, 4 h. Others: allylbenzene, isopropenylbenzene, 1,1'-(1,5-hexadiene-1,6-diyl)bisbenzene and other unknown by-products.

Table S5. Hydrogenation of CAL to COL over various catalytic systems.

Catalyst	Hydrogen donor	Temperature (°C)	Time (h)	Conv. (%)	Sel. (%)	Ref.
Pt/Co-Asp	H_2 2.0 MPa	60	2	89.7	89.3	[1]
Pt-Co/rGO	H_2 2.0 MPa	40	3	94.6	89.5	[2]
Pt-Co/SBA-15-0.6	H_2 1.0 MPa	80	2	71	91	[3]
Pt-Co/CNTs	H_2 1.5 MPa	60	1.5	77.7	93.6	[4]
Pt_8Co_1/ZSM-5-AT	H_2 2.0 MPa	100	2	＞99	65	[5]
$Fe_{0.5}Co$@NC	H_2 2.0 MPa	80	2	95.1	91.7	[6]
CoB-F-EG	H_2 1.0 MPa	100	4	94.9	84.9	[7]
CoPt/Fe_3O_4	H_2 3.0 MPa	160	4	95	84	[8]
Pt-Co/PCT	H_2 2.0 MPa	80	3	100	96	[9]
Co-Pt/MWCNTs	H_2 1.0 MPa	80	12	93.3	93.4	[10]
Pt_3-Co/rGO-MW	H_2 2.0 MPa	70	1.5	99.7	95.3	[11]
Pt-Co/N-CNT	H_2 2.0 MPa	70	1.5	99.7	87.9	[12]
$Pt_{50}Re_{50}$/rGO	H_2 2.0 MPa	120	4	94.1	88.7	[13]
Cu-MgO [a]	Isopropanol	180	7	11.6	100	[14]
Cu-MgO [a]	Cyclohexanol	100	7	97.2	100	[14]
UiO-66	Isopropanol	120	24	＞99	94	[15]
Co@CN-900	*n*-Hexanol	80	48	＞99	99	[16]
Co@CN-900	Isopropanol	80	48	97	90	[16]
Cu-Ru/MWCNT	$NaBH_4$	RT	0.5	100	69	[17]
Cu-Ru/MWCNT	Formic acid	100	1	44	73	[17]
Cu-Ru/MWCNT	Acetic acid	100	1	15	63	[17]
AuNPore	Et_3SiH	70	24	73	99	[18]
AuNPore	Formic acid	90	22	97	100	[19]
Co_1Re_1/TiO_2	Formic acid	140	4	99	89	This work

[a] 1 MPa N_2.

Table S6. Metal contents in fresh and spent Co_1Re_1/TiO_2 catalyst measured by ICP-OES.

Catalyst	Co (wt%)	Re (wt%)
Fresh Co_1Re_1/TiO_2	1.98	6.31
Spent Co_1Re_1/TiO_2	1.90	6.22

Table S7. Labels and calculated metal contents of catalysts.

Catalyst Label	Co (wt%)	Re (wt%)	Co/Re (mol/mol)
Co/TiO_2	2.0	0	N/A
Co_2Re_1/TiO_2	2.0	3.2	2.0
Co_1Re_1/TiO_2	2.0	6.3	1.0
Co_1Re_2/TiO_2	2.0	12.6	0.5
Re/TiO_2	0	2.0	0

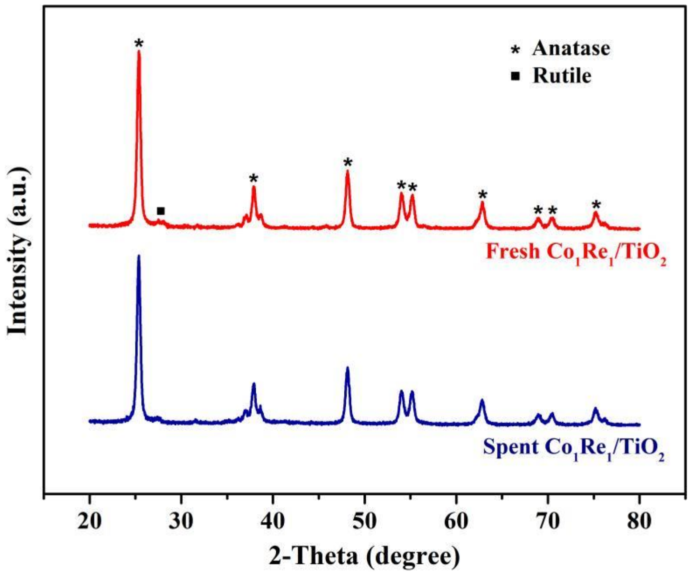

Figure S1. XRD patterns of fresh and spent Co_1Re_1/TiO_2.

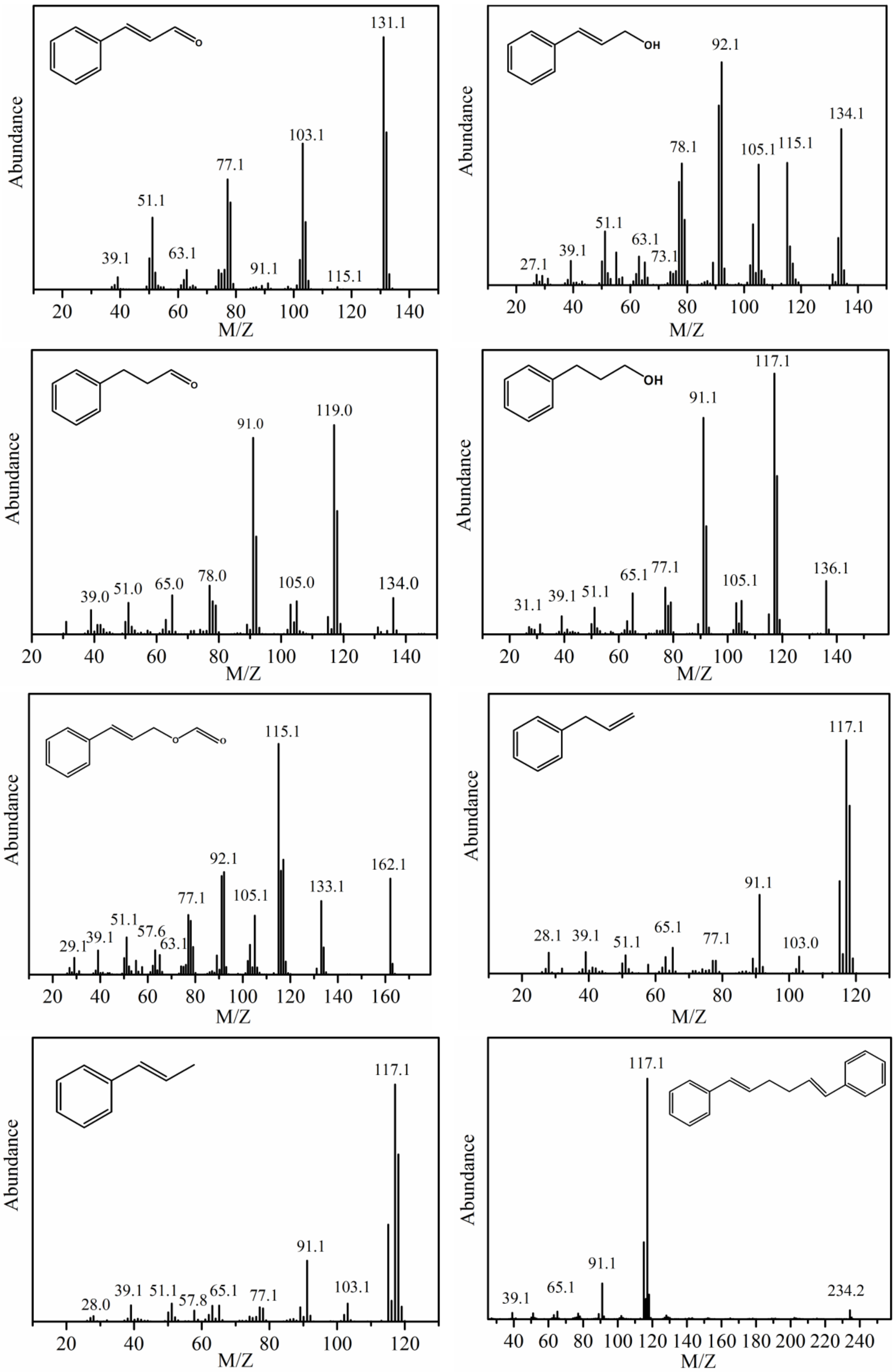

Figure S2. GC/MS spectra of substrate and products in the hydrogenation of COL.

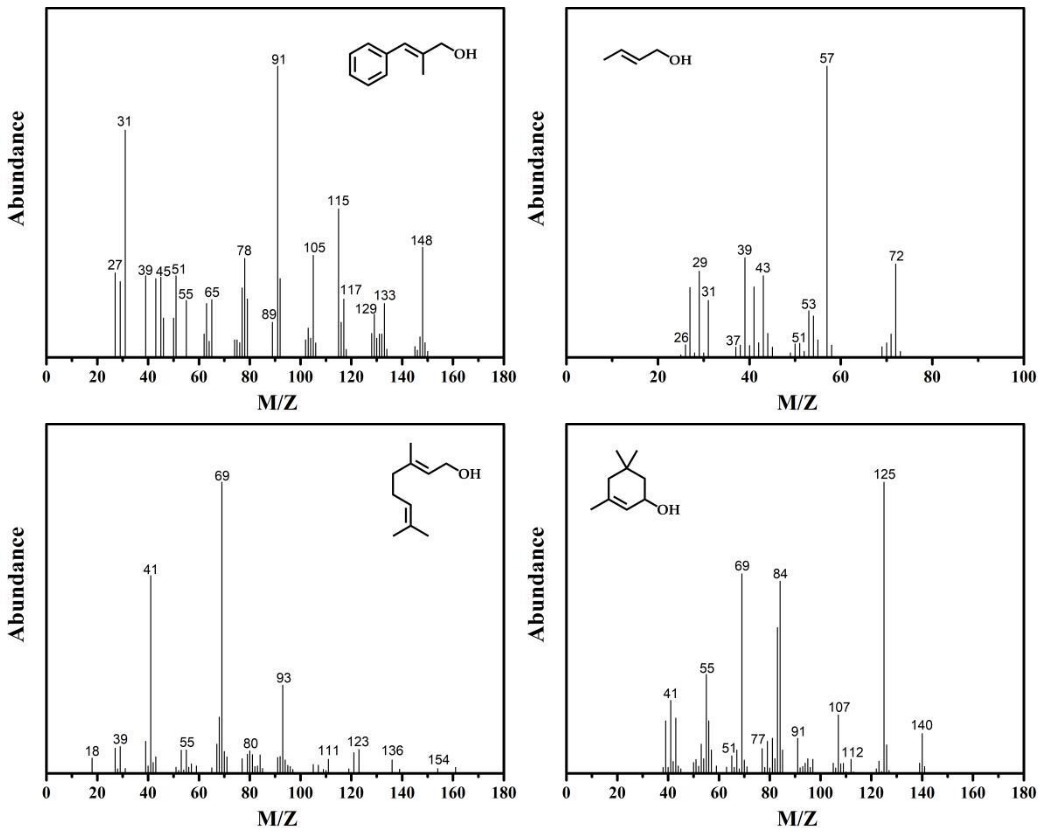

Figure S3. GC/MS spectra of the main products in the hydrogenation of other α,β-unsaturated aldehydes.

References

1. Tang Y.; Li H.; Cui K.; Xia Y.; Yuan G.; Feng J.; Xiong W. Chemoselective hydrogenation of cinnamaldehyde over amorphous coordination polymer supported Pt-Co bimetallic nanocatalyst. *Chem. Phys. Lett.* **2022**, *801*, 139683. https://doi.org/10.1016/j.cplett.2022.139683
2. Rong Z.; Sun Z.; Wang Y.; Lv J.; Wang Y. Selective hydrogenation of cinnamaldehyde to cinnamyl alcohol over graphene supported Pt–Co bimetallic catalysts. *Catal. Lett.* **2014**, *144*, 980-986. https://doi.org/10.1007/s10562-014-1209-5
3. Zheng Q.; Wang D.; Yuan F.; Han Q.; Dong Y.; Liu Y.; Niu X.; Zhu Y. An effective Co-promoted platinum of Co–Pt/SBA-15 catalyst for selective hydrogenation of cinnamaldehyde to cinnamyl alcohol. *Catal. Lett.* **2016**, *146*, 1535-1543. https://doi.org/10.1007/s10562-016-1779-5
4. Li Y.; Li Z.-G.; Zhou R.-X. Bimetallic Pt-Co catalysis on carbon nanotubes for the selective hydrogenation of cinnamaldehyde to cinnamyl alcohol: Preparation and characterization. *J. Mol. Catal. A: Chem.* **2008**, *279*, 140-146. https://doi.org/10.1016/j.molcata.2007.10.015
5. Cheng S.; Lu S.; Liu X.; Li G.; Wang F. Enhanced activity of alkali-treated ZSM-5 zeolite-supported Pt-Co catalyst for selective hydrogenation of cinnamaldehyde. *Molecules* **2023**, *28*, 1730. https://doi.org/10.3390/molecules28041730
6. Lv Y.; Han M.; Gong W.; Wang D.; Chen C.; Wang G.; Zhang H.; Zhao H. Fe-Co alloyed nanoparticles catalyzing efficient hydrogenation of cinnamaldehyde to cinnamyl alcohol in water. *Angew. Chem. Int. Ed. Engl.* **2020**, *59*, 23521-23526. https://doi.org/10.1002/anie.202009913
7. Zhao J.; Malgras V.; Na J.; Liang R.; Cai Y.; Kang Y.; Alshehri A. A.; Alzahrani K. A.; Alghamdi Y. G.; Asahi T.; Zhang D.; Jiang B.; Li H.; Yamauchi Y. Magnetically induced synthesis of mesoporous amorphous CoB nanochains for efficient selective hydrogenation of cinnamaldehyde to cinnamyl alcohol. *Chem. Eng. J.* **2020**, *398*, 125564. https://doi.org/10.1016/j.cej.2020.125564
8. Yuan T.; Liu D.; Pan Y.; Pu X.; Xia Y.; Wang J.; Xiong W. Magnetic anchored CoPt bimetallic nanoparticles as selective hydrogenation catalyst for cinnamaldehyde. *Catal. Lett.* **2018**, *149*, 851-859. https://doi.org/10.1007/s10562-018-2619-6
9. Gu Z.; Chen L.; Li X.; Chen L.; Zhang Y.; Duan C. NH_2-MIL-125(Ti)-derived porous cages of titanium oxides to support Pt-Co alloys for chemoselective hydrogenation reactions. *Chem. Sci.* **2019**, *10*, 2111-2117. https://doi.org/10.1039/c8sc05450a
10. Wang X.; He Y.; Liu Y.; Park J.; Liang X. Atomic layer deposited Pt-Co bimetallic catalysts for selective hydrogenation of α, β-unsaturated aldehydes to unsaturated alcohols. *J. Catal.* **2018**, *366*, 61-69. https://doi.org/10.1016/j.jcat.2018.07.031
11. Shi J.; Nie R.; Zhang M.; Zhao M.; Hou Z. Microwave-assisted fast fabrication of a nanosized Pt_3Co alloy on reduced graphene oxides. *Chin. J. Catal.* **2014**, *35*, 2029-2037. https://doi.org/10.1016/s1872-2067(14)60232-1
12. Tian Z.; Liu C.; Li Q.; Hou J.; Li Y.; Ai S. Nitrogen- and oxygen-functionalized carbon nanotubes supported Pt-based catalyst for the selective hydrogenation of cinnamaldehyde. *Appl. Catal. A-Gen.* **2015**, *506*, 134-142. https://doi.org/10.1016/j.apcata.2015.08.023

13. Wei Z.; Zhu X.; Liu X.; Xu H.; Li X.; Hou Y.; Liu Y. Pt-Re/rGO bimetallic catalyst for highly selective hydrogenation of cinnamaldehyde to cinnamylalcohol. *Chin. J. Chem. Eng.* **2019**, *27*, 369-378. https://doi.org/10.1016/j.cjche.2018.04.022
14. Siddqui N.; Sarkar B.; Pendem C.; khatun R.; Sivakumar Konthala L. N.; Sasaki T.; Bordoloi A.; Bal R. Highly selective transfer hydrogenation of α,β-unsaturated carbonyl compounds using Cu-based nanocatalysts. *Catal. Sci. Technol.* **2017**, *7*, 2828-2837. https://doi.org/10.1039/c7cy00989e
15. Plessers E.; De Vos D.E.; Roeffaers M.B.J. Chemoselective reduction of α,β-unsaturated carbonyl compounds with UiO-66 materials. *J. Catal.* **2016**, *340*, 136-143. https://doi.org/10.1016/j.jcat.2016.05.013
16. Liu X.; Cheng S.; Long J.; Zhang W.; Liu X.; Wei D. MOFs-derived Co@CN bi-functional catalysts for selective transfer hydrogenation of α,β-unsaturated aldehydes without use of base additives. *Mater. Chem. Front.* **2017**, *1*, 2005-2012. https://doi.org/10.1039/c7qm00189d
17. Hareesh H.N.; Minchitha K.U.; Venkatesh K.; Nagaraju N.; Kathyayini N. Environmentally benign selective hydrogenation of α,β-unsaturated aldehydes and reduction of aromatic nitro compounds using Cu based bimetallic nanoparticles supported on multiwalled carbon nanotubes and mesoporous carbon. *RSC Adv.* **2016**, *6*, 82359-82369. https://doi.org/10.1039/c6ra04241d
18. Takale B.S.; Wang S.; Zhang X.; Feng X.; Yu X.; Jin T.; Bao M.; Yamamoto Y. Chemoselective reduction of α,β-unsaturated aldehydes using an unsupported nanoporous gold catalyst. *Chem. Commun.* **2014**, *50*, 14401-14404. https://doi.org/10.1039/c4cc07068b
19. Butt M.; Feng X.; Yamamoto Y.; Almansour A.I.; Arumugam N.; Kumar R.S.; Bao M. Unsupported nanoporous Gold-catalyzed chemoselective reduction of α,β-unsaturated aldehydes using formic acid as hydrogen source. *Asian J. Org. Chem.* **2017**, *6*, 867-872. https://doi.org/10.1002/ajoc.201700130
